# Supplementary material for: Higher CSF sTNFR1-related proteins associate with better prognosis in very early Alzheimer’s disease
Source: Nat Commun. 2021 Jun 28;12:4001. doi: 10.1038/s41467-021-24220-7 (PMC8238986; doi:10.1038/s41467-021-24220-7)
Supplement: Supplementary file 1 — Supplementary Information [file 41467_2021_24220_MOESM1_ESM.pdf]

Supplementary Information

Higher CSF sTNFR1-related proteins associate with better prognosis in very early Alzheimer's disease

Authors: William T. Hu, Tugba Ozturk, Alexander Kollhoff, Whitney Wharton, J. Christina Howell, Alzheimer's Disease Neuroimaging Initiative
Corresponding author: William T. Hu, William.hu@rutgers.edu

Supplementary Methods

Cohort B (NCT02089555; PI: WTH) sought to recruit older White and Black American participants with NC, MCI, and AD dementia in Georgia. Inclusion criteria included: age 60-85 (inclusive); has normal cognition, a diagnosis of mild cognitive impairment, or a diagnosis of Alzheimer's disease; self-reported race of Black/African American or non-Hispanic white; able to undergo neuropsychological testing, lumbar puncture, and MRI; and English speaking. Exclusion criteria include: history of large territory stroke; diagnosis of Parkinson's disease, amyotrophic lateral sclerosis, or another progressive neurological disorder which may spare cognition; Mini-Mental State Examination score < 17.

Cohort C (NCT00135226; PI: WW) sought to recruit middle-aged to older white and Black participants with NC and family history of dementia in Georgia. Inclusion criteria included: age 45 – 65 (inclusive); a biological parent with AD dementia; willing to fast for eight hours; willing to undergo all procedures including LP. Exclusion Criteria: contraindication for LP; history of neurologic disease or significant head trauma; major untreated depression within two years; history of alcohol or substance abuse; any significant systemic illness or unstable medical condition which could affect cognition or cause difficulty complying with the protocol; diagnosis

of MCI, AD dementia, or residence in a skilled nursing facility; use of investigational medication; unwillingness to fast.

Supplementary Fig 1. Between-subject variability of cognitive decline in ADNI. Longitudinal CDR-SB changes up to 120 months following CSF collection according to clinical diagnosis of NC (a), MCI (b), and AD dementia (c)showed great between-subject variability even after incorporating predicted ADNC status (CSF t-Tau/Aβ42 levels relative to 0.39[1]).

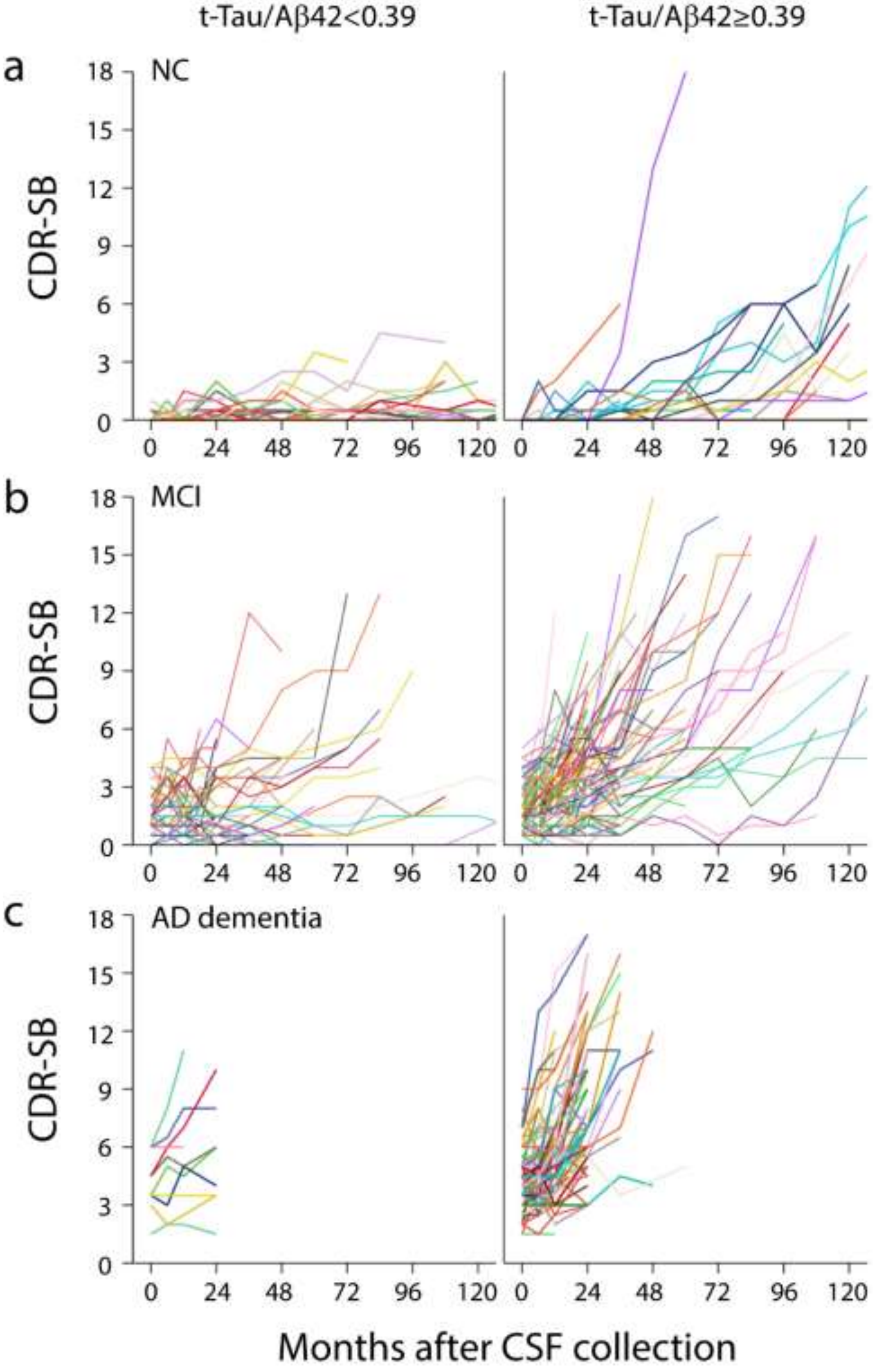

Supplementary Fig 2. Frequency of baseline CDR-SB scores according to baseline diagnosis.

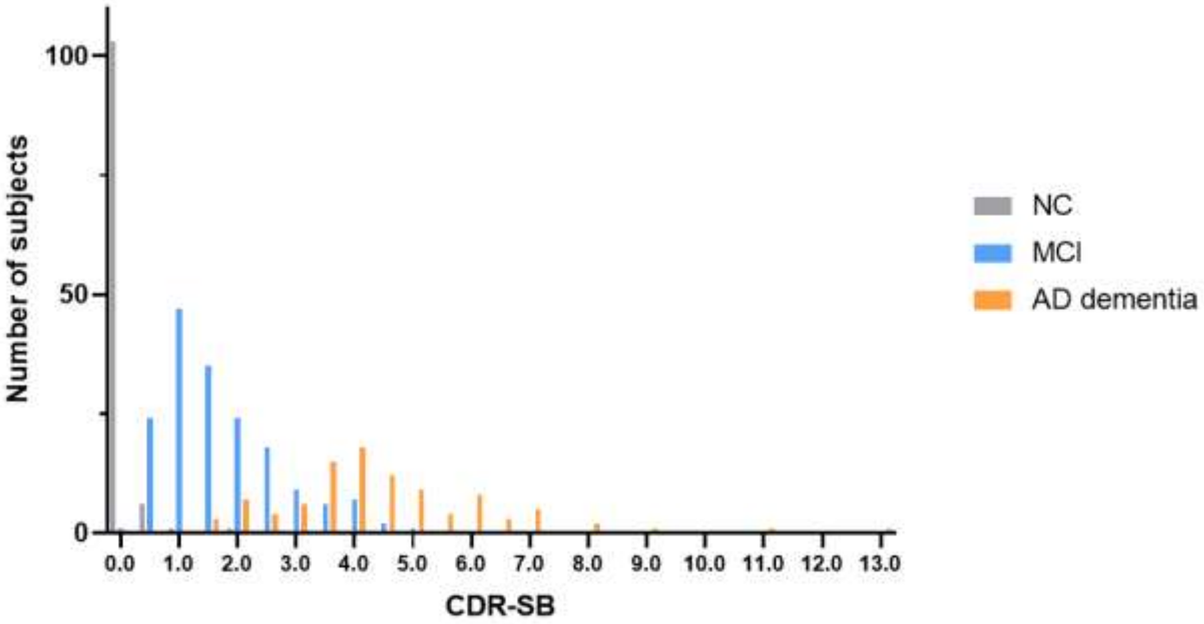

Supplementary Table 1. Linear mixed modeling of longitudinal CDR-SB changes according to basic baseline factors across the entire cohort. Significant interaction terms between Time (measured in months) and baseline factors are interpreted as baseline factors' effects on the CDR-SB slope of change over time.

	B (95% confidence interval)	P
Baseline diagnosis		
NC	Reference	
MCI	1.552 (1.223, 1.880)	<0.001
AD dementia	4.348 (3.938, 4.758)	<0.001
Baseline diagnosis X Months		
NC	Reference	
MCI	0.030 (0.001, 0.059)	0.043
AD dementia	0.076 (0.016, 0.137)	0.013
Age	0.002 (-0.016, 0.021)	0.793
Age X Months	0.002 (0.002, 0.003)	<0.001
Female sex	0.096 (-0.372, 0.180)	0.495
Female sex X Months	0.015 (-0.003, 0.034)	0.104
Education	0.032 (-0.013, 0.077)	0.166
Education X Months	0.002 (-0.001, 0.005)	0.201
Predicted ADNC	0.046 (-0.274, 0.367)	0.775
Predicted ADNC X Months	0.020 (-0.012, 0.052)	0.216
Predicted ADNC X Diagnosis X Months		
NC	Reference	
MCI	0.048(0.007, 0.089)	0.020
AD dementia	0.070 (0.001, 0.138)	0.045

Supplementary Table 2. Mean and standard deviation values for log_{10}-transformed cytokine values from ADNI NC participants.

	**Mean ± SD**
log_{10}(t-Tau)	1.808±0.172
log_{10}(p-Tau_{181})	1.344±0.202
log_{10}(MSD-sTREM2 MSD)	3.556±0.238
log_{10}(WU-sTREM2)	2451±762
log_{10}(MSD-GRN)	3.180±0.108
log_{10}(TNFα)	0.233±0.173
log_{10}(sTNFR1)	2.924±0.116
log_{10}(sTNFR2	2.999±0.132
log_{10}(sVCAM1)	4.569±0.199
log_{10}(sICAM1)	2.503±0.206
log_{10}(IL-6)	0.513±0.224
log_{10}(IL-7)	-0.068±0.432
log_{10}(IL-9)	0.513±0.224
log_{10}(IL-10)	0.722±0.187
log_{10}(IL12-p40)	-0.480±1.006
log_{10}(IL-21)	0.737±0.712
log_{10}(IP-10)	3.712±0.151
log_{10}(TGFβ1)	2.005±0.171
log_{10}(TGFβ2)	2.188±0.110
log_{10}(TGFβ3)	0.555±0.403

Supplementary Table 3. Relationship between CSF cytokine levels, clinical diagnosis, and CSF biomarker profile using ANCOVA adjusting for age, sex, and *APOE* ε4 status (F- and p-values are shown; p<0.01 used as threshold to adjust for multiple comparisons). Among the 15 CSF inflammatory proteins analyzed in this cohort, CSF levels of four analytes – sTNF-R1, sTNF-R2, TGF-β1, and sICAM1 – differed according to predicted ADNC status (p<0.001 for all), and TGF- β2 levels differed according to baseline diagnosis (p=0.006). As expected for the core CSF AD biomarkers, Aβ42 levels differed according to baseline diagnosis (p=0.002), and levels of all three markers (Aβ42, t-Tau, p-Tau$_{181}$, p<0.001) differed according to predicted ADNC status. Neither sTREM2 or progranulin levels varied according to baseline diagnosis or predicted ADNC status in this cohort.

	Diagnosis	t-Tau/Aβ42≥0.39	Diagnosis X t-Tau/Aβ42≥0.39
TNF-α	1.153, 0.317	1.598, 0.207	0.119, 0.888
sTNFR1	2.248, 0.107	17.473, <0.001	1.927, 0.147
sTNRF2	2.016, 0.135	22.298, <0.001	0.670, 0.513
IL-6	1.597, 0.204	0.003, 0.956	3.031, 0.050
IL-7	0.152, 0.859	2.995, 0.084	0.661, 0.517
IL-12p40	1.486, 0.228	0.314, 0.576	0.438, 0.646
IP-10	0.612, 0.543	3.747, 0.054	0.364, 0.695
IL-10	0.177, 0.838	0.071, 0.789	0.081, 0.923
IL-9	2.574, 0.078	6.647, 0.010	0.155, 0.856
IL-21	0.947, 0.389	3.892, 0.049	1.336, 0.264
TGFβ1	2.460, 0.087	12.845, <0.001	1.685, 0.187
TGFβ2	5.112, 0.006	3.944, 0.048	2.737, 0.066
TGFβ3	0.993, 0.372	2.896, 0.090	3.031, 0.049
sICAM1	1.313, 0.270	14.029, <0.001	0.242, 0.785
sVCAM1	0.024, 0.976	4.068, 0.044	2.543, 0.080
MSD-sTREM2	0.188, 0.829	1.082, 0.299	0.485, 0.616
WU-sTREM2	0.355, 0.701	2.532, 0.113	0.247, 0.782
progranulin	0.173, 0.841	0.277, 0.599	0.314, 0.730
Aβ42	6.321, 0.002	196.192, <0.001	2.414, 0.091
t-Tau	3.058, 0.048	208.161, <0.001	3.212, 0.041
p-Tau$_{181}$	1.411, 0.245	160.684, <0.001	3.024, 0.050

Supplementary Table 4. PCA of MCI participants from ADNI, showing loading (≥0.100) with missing values excluded (loading with missing values replaced with means in parentheses).

	PC1	PC2	PC3	PC4	PC5	PC6	PC7	PC8
t-Tau	0·871 (0.871)	0.356 (0.331)						
p-Tau$_{181}$	0·866 (0.906)	0.128 (0.102)						
Aβ42	-0·745 (-0.792)	0.343 (0.348)	-0.142	-0.141				0.118
t-Tau/Aβ42	0·675 (0.922)	0.244 (0.161)			0.123			
sTNFR1	0.137 (0.173)	0·903 (0.848)	0.281 (0.297)			0.144 (0.127)	0.124 (0.122)	
sTNRF2	0.255 (0.223)	0·883 (0.785)	0.343 (0.324)	(0.219)		(0.107)	0.226 (0.167)	
sVCAM1		0·869 (0.836)	0.202 (0.168)	0.165 (0.135)			0.161	0.124 (0.166)
sICAM1	0.333 (0.292)	0·467 (0.468)		(0.150)	-0.198	0.378 (0.211)	0.199 (0.126)	-0·624 (-0.581)
IP-10	-0.136 (-0.102)	0.186 (0.224)	0.255 (0.268)		-0.153 (-0.120)	(0.119)	0·705 (0.672)	0.100 (0.139)
MSD-sTREM2		0.471 (0.383)	0.883 (0.789)	(0.135)		0.199 (0.132)	0.151	
WU-sTREM2		0.263 (0.215)	0.882 (0.850)				0.212 (0.170)	-0.142
progranulin		0.588 (0.325)		0.530 (0.662)		(-0.206)	(0.126)	(0.174)
IL-6				0·799 (0.824)		0.109 (0.155)	(-0.118)	(-0.107)
IL-10	-0.118 (-0.119)	0.155 (0.125)	0.212 (0.150)	0·662 (0.725)	-0.158		0.459 (0.459)	
TGFβ2		(-0.188)			0·820 (0.903)		-0.150 (-0.124)	
TGFβ1	0.137	0.469 (0.510)			0·354 (0.694)	0.270	(0.134)	-0.112 (-0.159)
TGFβ3	0.176		-0.128 (-0.128)		0·538 (0.612)			
IL-7		-0.116		0.284 (0.283)	-0.103	0·810 (0.769)	0.245	0.393 (0.244)
TNF-α		0.378 (0.331)		0.157 (0.506)		0·429 (0.435)	0.610 (0.382)	
IL-9	(0.100)	0.288 (0.230)	(0.143)	(-0.195)		0·601 (0.739)	0.155 (0.199)	-0.105 (-0.120)
IL12-p40	0.123			(0.141)		0.223 (0.112)	0·772 (0.849)	
IL-21		0.153	(-0.112)			(0.106)		0·904 (0.924)

Supplementary Table 5. Demographic, clinical, and biomarker information for Cohorts B & C. * AD biomarkers were measured using Luminex (Alzbio3, Fujirebio Diagnostics, Malvern, PA) in Cohort B, and ELISA (InnoTest Fujirebio Diagnostics) in Cohort C. The absolute values are well-characterized to result from differences in antibody pairing and assay platforms, and ELISA measures are converted to equivalent Luminex measures using a validated conversion formula.[2] † A threshold of t-Tau/Aβ42 ≥0.39 for Luminex-derived measures based on a previous autopsy-derived series[1] was selected.

	Cohort B (n=126)	Cohort C (n=68)
Male (%)	56 (44%)	24 (35%)
Age, mean (SD)	70·0 (7·6)	58·9 (6·8)
Education, mean (SD)	15·7 (2·9)	N.A.
Race		
Asian (%)	0	0
Black/African American (%)	58 (46%)	21 (31%)
Non-Hispanic white (%)	68 (54%)	47 (69%)
Non-Hispanic (%)	126 (100%)	68 (100%)
Having at least one *APOE* ε4 allele (%)	64/124 (51%)	33 (48%)
Diagnosis<br>NC<br>MCI<br>AD dementia	 51 (40%)<br>50 (40%)<br>25 (20%)	 68 (100%)<br>0<br>0
CSF biomarkers		
Aβ42, mean (SD) in pg/mL	210·3 (133·8)	709·4 (186·7)*
t-Tau, mean (SD) in pg/mL	60·2 (42·6)	295·9 (161·9)*
p-Tau$_{181}$, mean (SD) in pg/mL	22·1 (11·8)	48·4 (20·5)*
t-Tau/Aβ42 consistent with AD† (%)	47 (37%)	8 (12%)*
sTREM2, mean (SD) in pg/mL	339·1 (115·6)	340·2 (116·2)
TNF-α, mean (SD) in pg/mL	2.13 (0·78)	1·17 (0·85)
sTNFR1, mean (SD) in pg/mL	622 (184)	570 (162)
sTNFR2, mean (SD) in pg/mL	878 (308)	706 (222)
TGFβ1, mean (SD) in pg/mL	N.D.	N.D.
TGFβ2, mean (SD) in pg/mL	N.D.	N.D.
TGFβ3, mean (SD) in pg/mL	N.D.	N.D.
IP-10, mean (SD) in ng/mL	3·61 (1·89)	N.D.
IL-6, mean (SD) in pg/mL	N.D.	N.D.
IL-7, mean (SD) in pg/mL	3·54 (2·13)	1·65 (0·79)
IL-9, mean (SD) in pg/mL	2·78 (1·97)	3·66 (2·06)
IL-10, mean (SD) in pg/mL	7·25 (3·60)	5·74 (2·52)
IL-12p40, mean (SD) in pg/mL	N.D.	N.D.
IL-21, mean (SD) in pg/mL	N.D.	N.D.
sICAM-1, mean (SD) in pg/mL	139·3 (88·0)	299·0 (160·0)
sVCAM-1, mean (SD) in ng/mL	18·2 (9·9)	24·9 (10·7)

Supplementary Table 6. MCI models of longitudinal ADNI-Mem-EF changes using biomarker family scores (from PCA), 0-60 months after CSF collection (ΔAIC=11.7, significant factors highlighted in blue with p<0.00625 used for CSF biomarkers to adjust for multiple comparisons).

	AIC = 386.4		AIC = 374.7	
	B (95% CI)	P	B (95% CI)	P
Months	0.013 (-0.012, 0.038)	0.298	0.028 (0.002, 0.054)	0.033
Baseline Cognitive Z	0.942 (0.890, 0.995)	<0.001	0.943 (0.911, 0.996)	<0.001
Baseline Cognitive Z X Months	0.0054 (0.0006, 0.0101)	0.027	0.004 (0, 0.009)	0.070
Female sex	0.011 (-0.053, 0.075)	0.734	0.013 (-0.052, 0.078)	0.695
Female sex X Months	-0.008 (-0.014, -0.003)	0.004	-0.006 (-0.012, 0.001)	0.019
Age	0 (-0.004, 0.004)	0.898	0 (-0.004, 0.005)	0.871
Age X Months	-0.0004 (-0.0008, 0.0001)	0.006	-0.0006 (-0.0009, -0.0003)	<0.001
APOE ε4+	0.075 (0.010, 0.140)	0.023	0.057 (0, 0.116)	0.060
AD score	-0.048 (-0.081, -0.015)	0.005	-0.046 (-0.080, -0.011)	0.009
AD score X Months	-0.006 (-0.009, -0.003)	<0.001	-0.008 (-0.011, -0.005)	<0.001
sTNFR1 score			-0.010 (-0.042, 0.022)	0.547
sTNFR1 score X Months			0.005 (0.002, 0.008)	<0.001
AD score X sTNFR1 score			0.007 (-0.026, 0.040)	0.667
AD score X sTNFR1 score X Months			0.002 (0, 0.005)	0.078

Supplementary Table 7. MCI models of longitudinal CDR-SB changes using biomarker scores (from PCA), 0-60 months after CSF collection (ΔAIC=8.2 for biomarker family scores when sTNFR1 score was introduced). Using p-Tau_{181} and sTNFR1 levels showed similar results (ΔAIC=16.5). Significant factors are highlighted in blue at p<0.00625 for CSF biomarker PC score/biomarkers to adjust for multiple comparisons).

	AIC=2842.3		AIC = 2834.1	
	B (95% CI)	P	B (95% CI)	P
Months	-0.094 (-0.203, 0.014)	0.090	-0.158 (-0.272, -0.045)	0.006
Baseline CDR-SB	0.912 (0.804, 1.019)	<0.001	0.906 (0.799, 1.013)	<0.001
Baseline CDR-SB X Months	0.013 (0.001, 0.026)	0.032	0.012 (0, 0.024)	0.059
Age	0.005 (-0.008, 0.018)	0.466	0.007 (-0.007, 0.022)	0.339
Age X Months	0.002 (0.001, 0.003)	0.003	0.003 (0.001, 0.004)	<0.001
Core AD score	-0.061 (-0.164, 0.041)	0.241	-0.060 (-0.163, 0.042)	0.246
Core AD score X Months	0.028 (0.016, 0.040)	<0.001	0.029 (0.017, 0.040)	<0.001
sTNFR1 score			-0.026 (-0.139, 0.086)	0.642
sTNFR1 score X Months			-0.020 (-0.033, -0.008)	0.002

	AIC=2836.2		AIC = 2819.7	
	B (95% CI)	P	B (95% CI)	P
Months	-0.126 (-0.238, -0.014)	0.028	-0.187 (-0.306, -0.068)	0.002
Baseline CDR-SB	0.903 (0.795, 1.010)	<0.001	0.905 (0.793, 1.016)	<0.001
Baseline CDR-SB X Months	0.017 (0.004, 0.030)	0.008	0.016 (0.004, 0.029)	0.010
Age	0.004 (-0.010, 0.018)	0.579	0.004 (-0.012, 0.020)	0.626
Age X Months	0.002 (0.001, 0.004)	0.002	0.003 (0.001, 0.004)	<0.001
APOE ε4+	-0.107 (-0.329, 0.115)	0.345	-0.106 (-0.336, 0.123)	0.363
APOE ε4+ X Months	0.032 (0.007, 0.058)	0.012	0.032 (0.007, 0.057)	0.011
$z\log_{10}$(p-Tau_{181})	-0.050 (-0.154, 0.054)	0.345	-0.047 (-0.158, 0.065)	0.411
$z\log_{10}$(p-Tau_{181}) X Months	0.009 (0.001, 0.017)	0.026	0.011 (0.003, 0.019)	0.007
$z\log_{10}$(sTNFR1)			0.014 (-0.115, 0.144)	0.825
$z\log_{10}$(sTNFR1) X Months			-0.021 (-0.034, -0.007)	0.004

Supplementary Table 8. AD dementia models of longitudinal cognitive decline using biomarker scores, with average ADNI-Mem-EF or CDR-SB as outcome, 0-36 months (ΔAIC=9.56 and 8.20; significant factors highlighted in blue with p<0.00625 used for CSF biomarkers to adjust for multiple comparisons).

	AIC = 92.29		AIC = 82.73	
	B (95% CI)	P	B (95% CI)	P
Months	-0.086 (-0.137, -0.036)	0.001	-0.070 (-0.121, -0.020)	0.007
Baseline ADNI-Mem-EF	0.999 (0.947, 1.051)	<0.001	0.997 (0.946, 1.047)	<0.001
Age	0.001 (-0.003, 0.006)	0.542	0.001 (-0.004, 0.005)	0.760
Age X Months	0.0008 (0.0002, 0.0015)	0.015	0.0006 (0, 0.0012)	0.070
APOE ε4+	0.079 (0.010, 0.149)	0.032	0.072 (0.004, 0.140)	0.038
Core AD biomarker score	-0.003 (-0.040, 0.033)	0.852	-0.004 (-0.040, 0.032)	0.832
Core AD biomarker score X Months	-0.003 (-0.008, 0.002)	0.177	-0.004 (-0.009, 0.001)	0.115
sTREM2 score			0.027 (-0.008, 0.062)	0.127
sTREM2 score X Months			0.006 (0.001, 0.011)	0.015

	AIC = 1181.0		AIC = 1172.8	
	B (95% CI)	P	B (95% CI)	P
Months	0.034 (-0.042, 0.110)	0.375	0.040 (-0.033, 0.111)	0.283
Baseline CDR-SB	0.968 (0.875, 1.060)	<0.001	0.968 (0.876, 1.061)	<0.001
Baseline CDR-SB X Months	0.028 (0.012, 0.045)	0.001	0.028 (0.012, 0.043)	0.001
Core AD biomarker score	-0.087 (-0.256, 0.081)	0.306	-0.091 (-0.259, 0.077)	0.285
Core AD biomarker score X Months	0.024 (-0.002, 0.050)	0.070	0.024 (0, 0.005)	0.050
sTREM2 score			0.004 (-0.162, 0.170)	0.964
sTREM2 score X Months			-0.040 (-0.065, -0.016)	0.001

Supplementary Table 9. NC model – ADNI-Mem-EF as outcome, 0-60 months (ΔAIC=6.0, significant factors highlighted in blue with p<0.00625 used for CSF biomarkers to adjust for multiple comparisons).

	AIC = 229.36		AIC = 223.35	
	B (95% CI)	P	B (95% CI)	P
Month	0.076 (0.017, 0.134)	0.011	0.102 (0.046, 0.158)	<0.001
$Month^2$	-0.001 (-0.002, 0)	0.025	-0.0016 (-0.0026, -0.0007)	<0.001
Baseline ADNI-Mem-EF	0.983 (0.889, 1,077)	<0.001	0.982 (0.889, 1.075)	<0.001
Baseline ADNI-Mem-EF X Month	-0.018 (-0.026, -0.010)	<0.001	-0.019 (-0.027, -0.011)	<0.001
Baseline ADNI-Mem-EF X $Month^2$	0.0004 (0.0002, 0.0005)	<0.001	0.0004 (0.0002, 0.0005)	<0.001
Age	-0.003 (-0.011, 0.005)	0.465	-0.002 (-0.011, 0.006)	0.541
Age X Month	-0.008 (-0.014, 0)	0.036	-0.001 (-0.002, 0)	0.004
Age X $Month^2$	1.0E-5 (-1.9E-6, 2.2E-5)	0.098	1.5E-5 (3.4E-6, 2.7E-5)	0.012
Male sex	-0.008 (-0.097, 0.081)	0.859		
Male sex X Months	0.007 (-0.001, 0.014)	0.093		
Male sex X $Months^2$	-0.0001 (-0.0002, 0)	0.065		
Core AD score	0.007 (-0.032, 0.046)	0.737	0.004 (-0.035, 0.044)	0.825
Core AD score x Month	-0.003 (-0.005, -0.002)	0.001	-0.003 (-0.005, -0.001)	0.001
IL6 score			-0.013 (-0.058, 0.030)	0.546
IL6 score X Month			0.005 (0.002, 0.009)	0.005
IL6 score X $Month^2$			-8.5E-5 (-1.4E-4, -3E-5)	0.004

Supplementary References

1 Shaw, L. M. *et al.* Cerebrospinal fluid biomarker signature in Alzheimer's disease neuroimaging initiative subjects. *Ann Neurol* **65**, 403-413, doi:10.1002/ana.21610 (2009).

2 Irwin, D. J. *et al.* Comparison of cerebrospinal fluid levels of tau and Abeta 1-42 in Alzheimer disease and frontotemporal degeneration using 2 analytical platforms. *Arch Neurol* **69**, 1018-1025, doi:10.1001/archneurol.2012.26 (2012).
