# Supplementary material for: Higher CSF sTNFR1-related proteins associate with better prognosis in very early Alzheimer’s disease
Source: Nat Commun. 2021 Jun 28;12:4001. doi: 10.1038/s41467-021-24220-7 (PMC8238986; doi:10.1038/s41467-021-24220-7)
Supplement: Supplementary file 3 — Reporting summary [file 41467_2021_24220_MOESM3_ESM.pdf]

nature research

Corresponding author(s): William T. Hu, MD, PhD

Last updated by author(s): Apr 19, 2021

Reporting Summary

Nature Research wishes to improve the reproducibility of the work that we publish. This form provides structure for consistency and transparency in reporting. For further information on Nature Research policies, see our Editorial Policies and the Editorial Policy Checklist.

Statistics

For all statistical analyses, confirm that the following items are present in the figure legend, table legend, main text, or Methods section.

n/a	Confirmed	
☐	☒	The exact sample size (*n*) for each experimental group/condition, given as a discrete number and unit of measurement
☐	☒	A statement on whether measurements were taken from distinct samples or whether the same sample was measured repeatedly
☐	☒	The statistical test(s) used AND whether they are one- or two-sided<br>*Only common tests should be described solely by name; describe more complex techniques in the Methods section.*
☐	☒	A description of all covariates tested
☐	☒	A description of any assumptions or corrections, such as tests of normality and adjustment for multiple comparisons
☐	☒	A full description of the statistical parameters including central tendency (e.g. means) or other basic estimates (e.g. regression coefficient) AND variation (e.g. standard deviation) or associated estimates of uncertainty (e.g. confidence intervals)
☐	☒	For null hypothesis testing, the test statistic (e.g. *F*, *t*, *r*) with confidence intervals, effect sizes, degrees of freedom and *P* value noted<br>*Give P values as exact values whenever suitable.*
☒	☐	For Bayesian analysis, information on the choice of priors and Markov chain Monte Carlo settings
☒	☐	For hierarchical and complex designs, identification of the appropriate level for tests and full reporting of outcomes
☐	☒	Estimates of effect sizes (e.g. Cohen's *d*, Pearson's *r*), indicating how they were calculated

Our web collection on statistics for biologists contains articles on many of the points above.

Software and code

Policy information about availability of computer code

Data collection	All statistical analyses were performed in SPSS 26.0
Data analysis	All statistical analyses were performed in SPSS 26.0

For manuscripts utilizing custom algorithms or software that are central to the research but not yet described in published literature, software must be made available to editors and reviewers. We strongly encourage code deposition in a community repository (e.g. GitHub). See the Nature Research guidelines for submitting code & software for further information.

Data

Policy information about availability of data

All manuscripts must include a data availability statement. This statement should provide the following information, where applicable:

- Accession codes, unique identifiers, or web links for publicly available datasets
- A list of figures that have associated raw data
- A description of any restrictions on data availability

All ADNI data (including CSF inflammatory protein measures) are available for public access at adni.loni.usc.edu contingent on adherence to the ADNI Data Use Agreement, and all data in the validation cohorts are available from the corresponding author on reasonable request.

Field-specific reporting

Life sciences study design

All studies must disclose on these points even when the disclosure is negative.

Sample size	A power calculation was performed to arrive at a total sample size of at least 250 (MCI and AD dementia together) to have a power of 0.95 to detect an effect size of 0.15 for fixed factors (sex, APOE4 status) and covariates (age, baseline cognitive function, two inflammatory protein levels, two core AD biomarker levels) in LMM of longitudinal cognitive decline with p=0.05 if the repeated measure correlation is 0.3, and has power of 0.83 to detect an effect size of 0.15 with p=0.02 if the repeated measure correlation is 0.2.
Data exclusions	No data exclusion.
Replication	Replication of PCA was successfully performed in two independent cohorts (B & C). Replication of core AD and sTNFR1 scores as prognostic markers was successful in an independent Atlanta cohort.
Randomization	Participants from ADNI were allocated into experimental groups based on length of longitudinal follow-up, availability of samples, and prior measures of CSF complement-related proteins. Co-variates including age, sex, and APOE genotype were controlled in linear mixed modeling. Samples were randomized during CSF inflammatory measurements.
Blinding	For CSF collection (through lumbar puncture), investigators were not blinded to the diagnosis. For ADNI CSF samples, all samples were blinded to identity during analysis, and investigators were only unblinded after measurements were uploaded to ADNI. For the Atlanta cohorts, CSF samples were deidentified and the samples were randomized across the plates according to diagnosis to avoid batch-related biases. Plate-loading was then performed with only CSF ID available but blinded to diagnosis and other potentially identifying information. CSF assays were then performed with only well location available but blinded to ID and the diagnosis until completion.

Reporting for specific materials, systems and methods

We require information from authors about some types of materials, experimental systems and methods used in many studies. Here, indicate whether each material, system or method listed is relevant to your study. If you are not sure if a list item applies to your research, read the appropriate section before selecting a response.

Materials & experimental systems

n/a	Involved in the study	
☒	☐	Antibodies
☒	☐	Eukaryotic cell lines
☒	☐	Palaeontology and archaeology
☒	☐	Animals and other organisms
☐	☒	Human research participants
☒	☐	Clinical data
☒	☐	Dual use research of concern

Methods

n/a	Involved in the study	
☒	☐	ChIP-seq
☒	☐	Flow cytometry
☒	☐	MRI-based neuroimaging

Human research participants

Policy information about studies involving human research participants

Population characteristics	The ADNI cohort included 382 subjects (111 with normal cognition, 174 with mild cognitive impairment, and 97 with dementia), including 225 men (59%), 365 non-Hispanic whites (95%), and median age of 75.6 (interquartile range 71.3 to 79.9). Refer to Table 1 and Supplementary Table 4 for detailed descriptions.
Recruitment	ADNI participants were recruited independently through participating sites in US. Cohort B (NCT02089555; PI: WTH) was a cohort of older White (n=68) and Black American (n=62) subjects with NC, MCI, and AD dementia recruited from Georgia who underwent detailed prospective baseline neurological, neuropsychological, CSF, and MRI characterization for identifying race-associated biomarker differences. Participants in this study were recruited from the Emory Cognitive Neurology Clinic, Emory Alzheimer's Disease Research Center, or community events in the greater Atlanta area. Cohort C (NCT00135226; PI: WW) was a cohort of middle-aged to older White (n=47) and Black subjects with NC (n=21) who underwent detailed prospective baseline and longitudinal neuropsychological, CSF, and MRI characterization for identifying effect of race on vascular and AD biomarkers. Participants in this study were recruited from the Emory Alzheimer's Disease Research Center, community events, or dementia caregiving groups in the greater Atlanta.<br><br>All participants who consent to longitudinal aging studies involving CSF and MRI have better general health, are not treated with chronic anti-coagulation, and do not have metal implants incompatible with MRI. This may bias the range of CSF protein distribution. However, because our study shows certain inflammatory proteins associated with opposite in vitro functions are positively correlated in CSF, these potential biases' direction and effect size need to be empirically determined (eg, hospitalized cohorts).
Ethics oversight	This study was approved by the Emory University Institutional Review Board, the National Institutes on Aging, and the ADNI Resource Allocation and Review Committee

Note that full information on the approval of the study protocol must also be provided in the manuscript.
